# Efficacy of a bystander intervention for preventing dating violence in Brazilian adolescents: short-term evaluation

**DOI:** 10.1186/s41155-019-0133-4

**Published:** 2019-10-16

**Authors:** Karine Brito dos Santos, Sheila Giardini Murta, Luis Gustavo do Amaral Vinha, Juliana Silva de Deus

**Affiliations:** 1 Collegiate of Psychology, UniAmérica University Center, Foz do Iguaçu, Paraná Brazil; 2 Institute of Psychology, University of Brasília, Brasilia, Brazil; 3 Department of Statistics, University of Brasília, Campus Darcy Ribeiro, Brasília, DF CEP 70910-900 Brazil

**Keywords:** Prevention, Dating violence, Bystander approach, Efficacy, Empathy

## Abstract

Peers are the preferred source of help for Brazilian adolescents who experience dating violence. However, they are not always the best informants for effective responses for dealing with situations of violence in romantic relationships among peers. This experimental study aimed to evaluate the short-term efficacy of three aspects of a peer- and bystander approach-based intervention: the intent to offer help, empathy, and bystander attitudes in response to dating violence in a Brazilian sample of adolescents. The study’s participants were 33 adolescents randomized in two groups: experimental group (EG, *n* = 14) and control group (CG, *n* = 19). The EG underwent three weekly intervention sessions of 90 min each on the healthy versus violent romantic relationships, the quality of friendship in the peer network, and the role of the bystander, while the CG received no intervention. Evaluations were performed 1 week before and two and half months after the intervention. Statistically significant differences between EG and CG at post-test were not found in intention to help, empathy, and bystander attitudes. Future studies should include evaluations of larger samples and mid- and long-term follow-ups to identify patterns of change over the long term as well as examine barriers to utilization of bystander behaviors by adolescents in Brazilian culture.

## Background

Dating violence is a public health problem of great magnitude in Brazil (Barreira, De Lima, & Avanci, 2013; Flake, Barros, Schraiber, & Menezes, 2013; Oliveira, Assis, de Njaine, & Pires, 2014; Oliveira, de Assis, Njaine, & de Oliveira, 2011) and in the world (Leen et al., 2013; Niolon et al., 2015). According to the first epidemiological study about violence in dating and “flings” between Brazilian youth, 86.9% of adolescents have been victims of and 86.8% have been perpetrators of some form of violence, physical, sexual, or psychological, in their current or previous relationships (Oliveira et al., 2011). The negative impact of dating violence on mental and physical health is clear (Ellis, Crooks, & Wolfe, 2009; Foshee, Bauman, Linder, Rice, & Wilcher, 2007; Stader, 2011). Dating violence victimization has been associated with lower educational performance, decreased attachment to school, and school abandonment. A number of mental health concerns have been observed as well as a greater propensity for risky behaviors, such as alcohol and other drug abuse, depression, suicidal thoughts, and low levels of parental and neighborhood social support (Banyard & Cross, 2008). For Brazilian youth, other aspects that increase the vulnerability of adolescents involved in dating violence are related to sexually transmissible diseases, teen pregnancy, suicide, and death by homicide (Barreira, Lima, Bigras, Njaine, & Assis, 2014).

Risk and protection factors for the perpetration of dating violence were identified in a literature review of longitudinal studies (Vagi et al., 2013). Peer dominance is a prominent item in the list of relational violence factors, accounting for one third of them, and at least three other factors involve aspects of the friendship network, such as having friends involved in dating violence perpetration (Arriaga & Foshee, 2004; Foshee, Reyes, & Ennett, 2010), having friends who were victims of dating violence (Foshee et al., 2010), and low friendship quality (Linder & Collins, 2005). Among the protective factors, elevated empathy is highlighted by Vagi et al. (2013) at the individual level (McCloskey & Lichter, 2003). The identification of the sources of influence is fundamental to determining dating violence predictor factors that can be changed by means of an intervention (Foshee et al., 2010).

The literature recommends peer-based interventions that have several types of violence present in the peers’ world and dating in scope (Jenkins & Nickerson, 2017; Kettrey & Marx, 2018; McMahon & Banyard, 2012; Ramirez, Paik, Sanchagrin, & Heimer, 2012). The peers, while the primary source of normative influence and help for adolescents (Foshee et al., 2005), play a crucial role in the formation of romantic relationships, in their development and maintenance, and in protection during violent situations (Adams & Williams, 2011). Among Brazilian adolescents, peers are the preferential source of help for victims of violent intimate partners (Soares, Lopes, & Njaine, 2013). Although a strong influence in modeling behaviors and attitudes (Garrido & Taussig, 2013), adolescents may offer help that is not useful for dealing with the problem in an effective and safe manner (Weisz & Black, 2008). Evidence from a qualitative study with Brazilian youth has indicated that they are afraid of potential damage by offering inadequate or ineffective help due to a lack of skills (Murta, Ramos, Cangussú, Tavares, & Costa, 2014). Therefore, helping peers to competently intervene when they witness dating violence is a relevant preventive goal.

Emerging evidence places the role of the bystander at the center of efforts to prevent dating violence in North America (Amar, Tuccinardi, Heislein, & Simpson, 2015; Branch, Richards, & Dretsch, 2013; Coker et al., 2016; Cook-Craig et al., 2014; Jaime et al., 2014; Jaime et al., 2018; Palm Reed, Hines, Armstrong, & Cameron, 2014; Peterson et al., 2018); meanwhile, in Brazil, this approach has only recently been discussed (Santos & Murta, 2016) and used (Santos & Murta, in press) in the prevention of dating violence. Bystander-based programs share the philosophy that the change of social rules to prevent violence requires mobilizing the whole community (Coker et al., 2011) and seeks to transform community norms in the face of violence by means of strengthening the willingness, sense of responsibility, and skills for intervening of those witnessing episodes of violence (Cook-Craig et al., 2014). Approaching bystanders as preventive agents capable of intervening in dating violence is a potentially powerful tool, an alternative to programs focused on potential victims and aggressors. Upon attracting the peers as potential helpers, defensiveness in programs focused on the bystander tends to be lower, while the responsiveness to the messages of these programs tends to be higher (Burn, 2009).

Originally, the situational model of bystander intervention determined whether a bystander would intervene in an emergency by estimating the likelihood that a victim will receive help when in need (Darley & Latané, 1968). The model presupposed that before an individual decides to intervene, implicitly or explicitly, there are several preliminary steps to be followed, and at each step, the bystander may fail to help, which is why adopting a passive or active stance depends on how the circumstances are interpreted and how the bystander reacts to the underlying socially influential processes (Latané & Darley, 1968). Making the decision to intervene is a complex process involving five steps: (1) perceiving the situation (has the bystander noticed something is going on?), (2) interpreting it as an emergency (has the situation been correctly interpreted?), (3) assuming the responsibility to act (does the bystander see her/himself as responsible for doing something to help?), (4) deciding to act (has the bystander decided what to do?), and (5) acting to intervene (has the bystander engaged in the action) (Latané & Darley, 1970).

Although theoretical models centered on spectator help have long been available and practical applications in various contexts have been developed (Fischer et al., 2011), interventions based on the bystander approach for preventing violence in affective-sexual relationships remain of very recent vintage (Amar et al., 2015; Borsky, Mcdonnell, Turner, & Rimal, 2018; Coker et al., 2011; Coker et al., 2016; Coker, Bush, Brancato, Clear, & Recktenwald, 2018; Jaime et al., 2018; Miller et al., 2012; Palm Reed at al., 2015; Peterson et al., 2018). A systematic review of the literature (Storer, Casey, & Herrenkohl, 2016) identified nine programs with this purpose, and of them, only three sought to prevent dating violence, while the others focused exclusively on sexual violence not associated with dating. Moreover, all the studies identified were published in the last decade, which underscores their innovative character.

Evidence indicates the efficacy of bystander intervention to prevent dating violence via increasing the recognition of violence (Miller et al., 2012), the intention to help (Amar et al., 2015; Miller et al., 2012; Peterson et al., 2018), the perception of responsibility to help, the ability to offer help as bystanders (Amar et al., 2015), and helpful behaviors (Coker et al., 2011; Miller et al., 2012). Beyond this, the findings of these studies reveal a reduction in norms of acceptance of violence (Amar et al., 2015; Coker et al., 2011; Coker et al., 2018; Palm Reed et al., 2014; Peterson et al., 2018), interpersonal violence victimization and perpetration (Coker et al., 2015; Coker et al., 2016), sexual coercion, sexual harassment, stalking, and psychological dating violence victimization and perpetration (Coker et al., 2016). In addition to such effects at the individual level, changes at the community level are equally found, with reductions in the acceptance of dating and sexual violence in the school setting (Coker et al., 2018), dating violence victimization and perpetration, victimization and perpetration of sexual violence, sexual harassment, and stalking over the 5-year implementation of the preventive program in high schools (Coker et al., 2017).

Nevertheless, such positive evidence contrasts with the findings of other studies which have shown a lack of efficacy regarding the intent to help, self-efficacy, social norms, attitudes related to dating violence (Borsky et al., 2018), rape myth acceptance, and utilization of bystander behaviors (Moynihan et al., 2011). Furthermore, mixed results on utilization of bystander behaviors (Katz, Heisterkamp, & Fleming, 2011; Miller et al., 2012) and willingness to intervene (Miller et al., 2012) have been found. These results suggest that the development and evaluation of bystander-based programs for preventing dating violence compose a large domain of research still to be explored, within which dwell questions related to its efficacy to produce change at the individual and community levels as well as the context and mechanisms which generate such results.

### The present study

Although the adolescents frequently consult with peers when uncomfortable situations arise (Martsolf, Draucker, Bednarz, & Lea, 2011) and tend to reveal their dating violence experiences (Rizzo, 2009; Soares et al., 2013), many times they lack the ability to help friends with dating violence problems. In general, adolescents act less like counselors and more like confidantes, since although they live similar problems, they are not always able to help with the difficulties experienced by peers (Njaine, Oliveira, Ribeiro, de Minayo, & Bodstein, 2011; Weisz & Black, 2008; Weisz & Black, 2010). Teaching them to respond appropriately to the difficulties they share with friends is, thus, a promising path in preventing dating violence, especially if behaviors of seeking and offering help are spread in the friendship network.

**Fig. 1:**
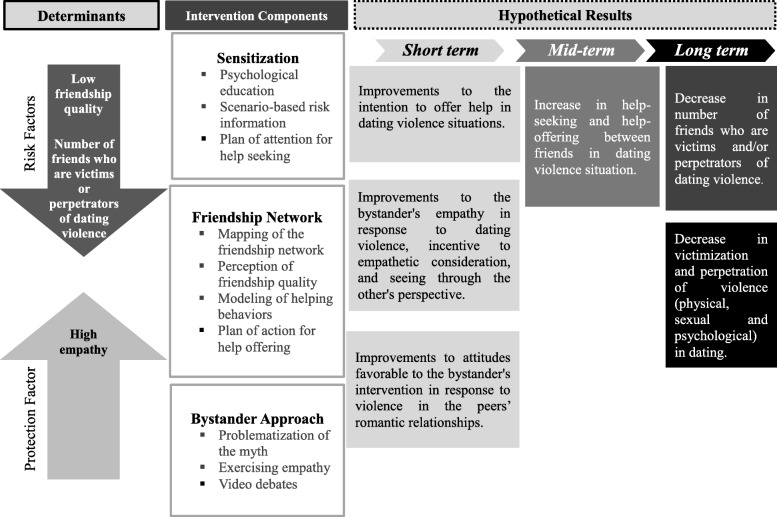
Intervention logic model

The current study extends prior research by examining the short-term effects of a peer- and bystander approach-based intervention to improve the intent to offer help, empathy, and bystander attitude outcomes at the level of the individual in response to dating violence in a Brazilian sample of adolescents. While the intention to help (Amar et al., 2015; Borsky et al., 2018; Miller et al., 2012; Peterson et al., 2018) and bystander attitudes (Amar et al., 2015) have been investigated in previous studies, empathy has been poorly explored in studies evaluating bystander interventions for preventing dating violence, though there is evidence for it as one of the protection factors for dating violence (McCloskey & Lichter, 2003) and it is considered an outcome of interest in bystander interventions in other types of violence (Jenkins & Nickerson, 2017). These short-term outcomes were considered precursors of medium-term (help-seeking and help-offering between friends in dating violence situation and friendship quality) and long-term (number of friends who are perpetrators and victims of dating violence and victimization and perpetration of dating violence) outcomes (Fig. 1), whose evaluation is outside the scope of this study. This study aimed to perform intra- and intergroup analysis comparing the effects of the intervention over each of the short-term outcomes cited above, 1 week before and two and half months after the intervention. The following hypotheses guided the study:

Hypothesis 1: Participants in the intervention group will more frequently present intention to help than participants in the control group at two and half months after the intervention.

Hypothesis 2: Participants in the intervention group will have higher scores than participants in the control group on empathy at two and half months after the intervention.

Hypothesis 3: Participants in the intervention group will have higher scores than participants in the control group on bystander attitudes in response to dating violence at two and half months after the intervention.

## Method

### Study design

**Fig. 2:**
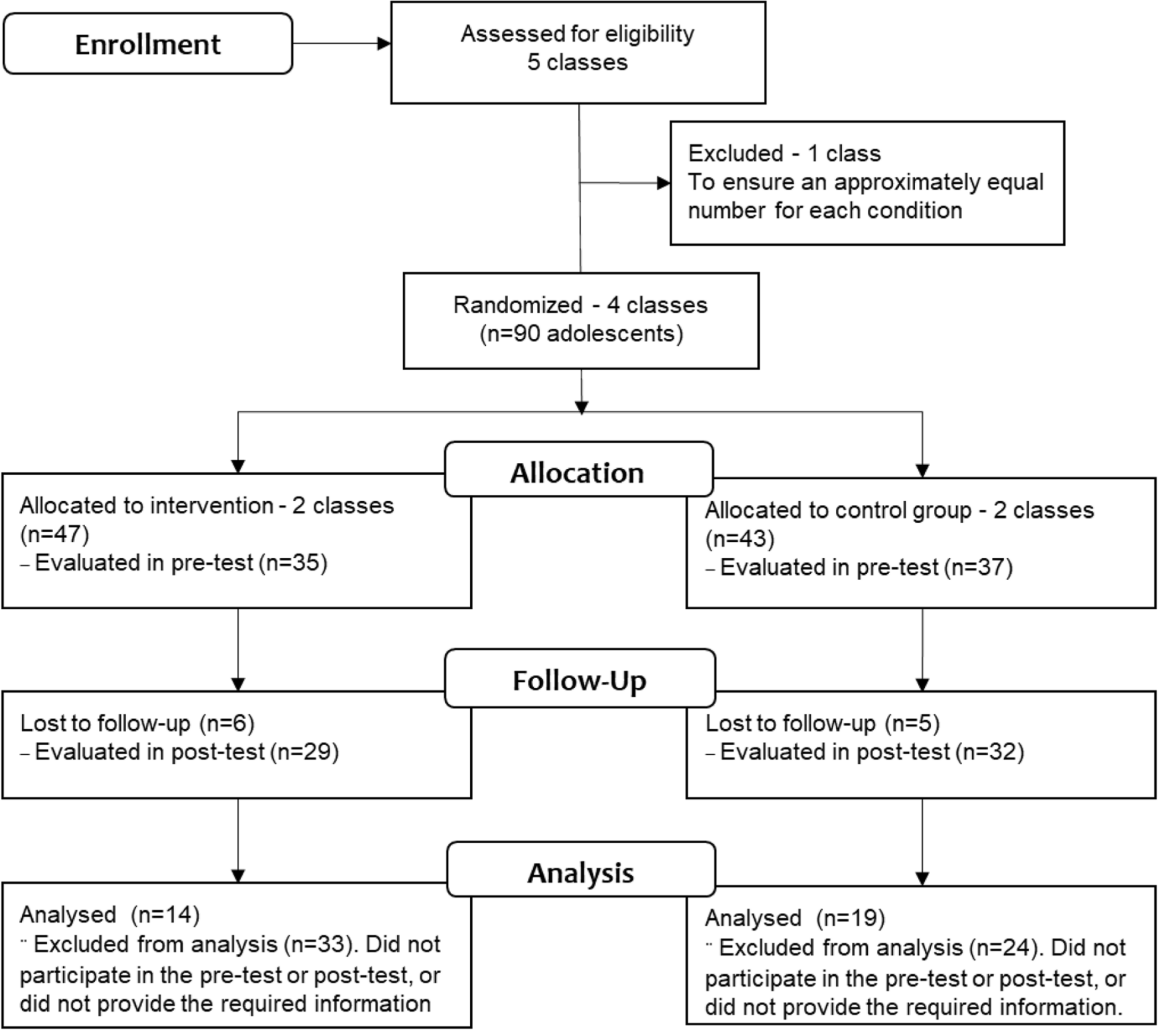
Flowchart of participants during each stage of the study

An experimental design, with pre- and post-test evaluations, the latter two and a half months after the intervention, and with an experimental (intervention) group (EG) and a control group (CG), whose adolescent participants were randomly assigned (Kazdin, 2010), was used. The participants were recruited from classes of the project “First Step to Work Program,” developed by the Socio-Professional Education Sector of one educational institution of the city of Brasília, Brazil. This project promotes the civic, personal, and profession education of socially vulnerable adolescents. Program classes, not individual students, were randomly selected to participate. Five classes were initially available for the study. One class was excluded to ensure an approximately equal number of participants for each experimental condition (two classes each) (Fig. 2).

### Participants

The participants comprised of 33 students, 14 in the experimental group (EG) and 19 in the control group (CG). Of a total of 47 students allocated in the EG, 35 were evaluated in the pre-test and 29 in the post-test, only 14 students with full data set. For the CG, 43 students were initially allocated, 37 were in pre-test, 32 were in post-test, and only 19 were in pre- and post-test and provided the required information. The inclusion criteria were age (at least 14 but less than 18 years of age) and voluntary participation. Possessing prior romantic experience with “flings” or “steady” relationships was not an inclusion criterion.

### Instruments

*Sociodemographic Questionnaire* consists of seven closed questions to collect age, gender, socioeconomic status, education level, race, religion, and romantic relationship status.

*Bystander Attitude in Dating Violence Scale* (*Escala de Atitudes do Espectador em Situações de Violência no Namoro*, ESPECTA-VN) is a Likert scale composed of 24 items developed to measure a bystander’s attitude regarding offering help in situations of physical, sexual, and psychological dating violence (Santos, 2016). The scale presents five factors which evaluate the following steps and respective barriers to the bystander’s intervention according to a model recommended by Latané and Darley (1970) with adaptations by Burn (2009): (1) awareness—failure to perceive the situation, (2) definition—failure to identify the risk involved in the situation, (3) responsibility—failure to take responsibility, (4) plan/self-efficacy—failure due to lack of abilities, and (5) acting to intervene—failure due to the audience effect. It is a 5-point scale ranging from completely disagree to completely agree (alphas = 0.78 to 0.96). Higher scores indicated greater likelihood for help from the bystander and thus lower propensity to fail to act having the following situational factors in play: sensorial distractions, pluralistic ignorance, attribution of merit due to use of alcohol/illicit drugs and provocative character of the partners, bystander’s relationship with the potential victim and potential aggressor, ignorance regarding the action and of how to act as a bystander, and preoccupation with the negative evaluation of other bystanders.

*Davis Multidimensional Interpersonal Reactivity Scale* (DMIRS—Davis, 1983), adapted for Brazilian samples by Koller, Camino, and Ribeiro (2001), was used to assess the participants’ empathy levels. The instrument has 21 items arranged in 3 subscales that evaluate respectively: (1) empathetic consideration—the affective components of empathy using items that reflect concern for other people; (2) taking the other’s perspective—the cognitive components of empathy, based in the adoption of another’s perspective and anticipation of their reactions; and (3) personal distress—the reactions of the individual in the face of the suffering of others in tense emotional situations. It is a 5-point Likert scale ranging from does not describe me well to describes me perfectly (alphas = 0.54 to 0.74).

*Intention to Help in Dating Violence Questionnaire* is composed of one closed question to evaluate intention to help (Santos, 2016). The question addressed the intention to help in a hypothetical situation (Would you get involved and offer help to a couple who was in a dating violence situation?), with “yes” or “no” as possible answers.

### Procedures

#### Intervention design

The given empirical evidence combined with various theories arising from distinct fields of knowledge serves as the theoretical and methodological basis for the intervention, including the bioecological model (Bronfenbrenner, 1996, Bronfenbrenner, 2004; Bronfenbrenner & Evans, 2000; Poletto & Koller, 2008), the cognitive social theory (Bandura, 1986), the social network theory (Sluzki, 1997), and the bystander intervention model (Latané & Darley, 1970). A synthesis of the change mechanisms proposed in this intervention is graphically depicted in Fig. 1. The intervention design along with a detailed description of the procedures was described elsewhere (Santos & Murta, 2017).

The intervention is composed of three weekly sessions of 90 min each. Table 1 presents the objectives, activities, and contents by session. The sessions comprise five steps: homework discussion (sessions 2 and 3 only), the day’s thematic approach, activity discussion, homework (sessions 1 and 2 only), and session evaluation. Each participant received an intervention support guide (Murta et al., 2011), whose contents were related to information on dating violence and life skills, which serve to bolster the recognition of violence and the seeking and offering of help in adolescents in violent situations with their own dates as well as among their peers. The intervention was conducted by a doctoral student in clinical psychology with broad training in group interventions and in the topics of dating violent prevention, peer intervention, and spectator approach.

**Table 1:** General overview of the intervention

Session	Objectives	Activities	Content
1. The two faces of dating	Discuss the relationship’s characteristics, differentiate healthy and unhealthy relationships. Raise awareness about the nature, dynamics, prevalence, causes, and consequences of dating violence in health.	Dynamic, playful, and interpretational reading of a comic book story. Evaluation of the relationship’s quality. (Murta et al., 2011, p. 40–46 e 59).	Modalities of intimate relationships between friends: one-night stands, flings, long-term relationships. Characteristics of dating relationships: intrinsic rewards (intimate self-revelation, care noticed from the partner), standards of influence and interaction (time spent with the partner, sexual intimacy, perceptions of balance and power), and problematic characteristics (jealousy, betrayal, lack of support to the partner, conflicts). Warning signs for dating violence.
2. Friendship network	Map the network of close friendships to name and visualize the friendship network as well as make it more tangible, improving the odds of mobilizing the help network. Identify positive and negative peer influences in the friendship network. Foster the functions of social/emotional support, cognitive guidance, and counseling in the friendship network.	Construction of the network map focused on close friendships	The role of friends in the emergence, development, and maintenance of dating relationships and in protection in cases of violence. Changes in the peer network and in the nature of relationships with friends as the relationship emerges. - Network structural characteristics: size, density, composition, dispersion, homo/heterogeneity. - Functions in the network: social company, cognitive and counseling guidance, social regulation, material help, help from services, and access to new contacts. - Attributes of the connection: predominant function, versatility, reciprocity, intensity/commitment, frequency of contact, and history. - Friendship functions: help, reliable alliance, self-validation, companionship, intimacy, and emotional security. Rules of peers that oppose the bystander’s intervention and associated gender roles.
3. Bystander approach	To undermine the myth that “a couple’s fight is no one else’s business,” encourage the adolescents to adopt attitudes favorable to intervening. To boost the modeling of helping behaviors in the friendship network, and to mobilize helping behaviors in the friendship network. To teach empathy skills to incentivize the adoption of empathetic communication and taking the other’s perspective in response to dating violence.	Video debate about the bystander approach intervention Exercising empathy	Friends as potential bystanders in dating conflict situations and as preferred sources of help. Roles (victim, aggressor, bystander) Stages of the bystander’s intervention: 1. Awareness 2. Definition 3. Responsibility 4. Plan/self-efficacy 5. Action Obstacles to the intervention How to stop being a “passive bystander” and start being an “active bystander.”

#### Data collection procedures

The pre-test was given a week before the first session, and the post-test 2 months after the last session. Participants were invited to answer the *Bystander Attitude in Dating Violence Scale*, *Davis Multidimensional Interpersonal Reactivity Scale*, and *Intention to Help in Dating Violence Questionnaire* before and after the intervention, while the *Sociodemographic Questionnaire* was only given in the pre-test. The questionnaires were filled out collectively in the classroom.

#### Data analysis procedures

Quantitative data analysis was performed using techniques of inferential and descriptive statistics. A per-protocol analysis was adopted given the aim of evaluating the intervention’s short-term effect under optimal conditions, defined for the purpose of this study as attendance to the intervention and answering the pre-test and post-test evaluations. Participants who abandoned the intervention and did not provide data in both evaluations were eliminated from data analysis. For comparisons of EG and CG group participants at baseline, chi-squared and Fisher’s exact tests were used. The Wilcoxon signed-rank test and Cohen *d* were used to analyze in group differences. The comparison between the groups’ pre-test and post-test differences was performed using the Kruskall-Wallis test. Intention to help changes were analyzed using McNemar’s test. The significance level adopted for all statistical tests was 5%. The analyses were done using SPSS (Statistical Package for the Social Sciences), version 18.

### Ethical aspects

This study has been approved by the Committee of Research Ethics of the Institute of Research of Human Sciences of the University of Brasília (opinion n. ° 411.000). The parents and adolescents were informed by written and oral means about the voluntary participation, research objectives, the confidentiality of personal data, the use of the collected information, and the freedom to cease participating in the study at any moment. The parents and adolescents who agreed to participate in the study signed the terms of informed consent (parents) or the terms of informed assent (adolescents). Consent from the educational institution was also obtained.

## Results

### Intergroup baseline comparison

Table 2 presents the sociodemographic profile of the participants. The majority of the participants were female, 64.3% and 52.6% in the EG and CG, respectively. All the participants were public education enrollees and, in general, possessed a partial high school education (EG = 85.7% and CG = 100%). The majority of both groups were 16 or 17 years old. The socioeconomic level was higher for the control group: 78.9% of the CG students and 71.4% of the EG students were classified in classes A, B1, and B2 of the Brazilian Economic Classification Criteria (Associação Brasileira de Empresas de Pesquisa, 2014). In both groups, the greater part of the pupils self-reported as being brown-skinned (EG = 50.0% and CG = 63.2%). The plurality declared themselves to be religiously Evangelical (EG = 42.9% and CG = 52.6%), followed by Catholic (EG = 35.7% and CG = 31.6%). At the time of the study, many of the students reported not being in a relationship (EG = 71.4% and CG = 42.1%), with 28.6% and 31.6% respectively declaring themselves to be in a relationship. As for marital status, a greater percentage of the experimental group youths were single (92.9%) as compared to the control youths (78.9%). There was no statistically significant difference between groups for these sociodemographic variables.

**Table 2:** Participants’ profile

Variable	Group		
	Experimental (*n* = 14)	Control (*n* = 19)	*p* value
Gender			
Female	64.3%	52.6%	0.754
Male	35.7%	47.4%	
Education			
Partial high school	85.7%	100%	0.336
Completed high school	14.3%	0%	
Age (years)			
16	33.3%	61.1%	0.116
17	50.0%	38.9%	
18	16.7%	0.0%	
Social-economic level			
Class A	7.1%	31.6%	0.411
Class B1	14.3%	10.5%	
Class B2	50.0%	36.8%	
Class C1	28.6%	21.1%	
Race			
White	14.3%	21.1%	0.586
Black	28.6%	10.5%	
Yellow	7.1%	5.2%	
Brown	50.0%	63.2%	
Religion			
None	21.4%	15.8%	0.843
Catholic	35.7%	31.6%	
Evangelical	42.9%	52.6%	
Relationship status			
Currently single	71.4%	42.1%	0.173
Occasional dates with different people	0.0%	10.5%	
Occasional dates with the same person	0.0%	15.7%	
Stable/long-term relationship	28.6%	31.6%	
Marital status			
Single	92.9%	78.9%	0.624
Stable union	7.1%	10.5%	
Married	0.0%	10.6%	

### Intragroup comparison of experimental data

Half of the EG participants (50%) answered yes to the question about the intention to offer help to couples in situations of violence before the intervention (Table 3). After the intervention, there was an increase in intention (86.0%), but this difference was not significant (*p* > 0.05). The analysis of intraindividual change in intention to help in the EG showed that seven participants, who declared they would not help on pre-test, said they would help on the post-test. The opposite was verified for only two participants. There was also a non-significant increase in intention to offer help in the CG (*p* > 0.05).

**Table 3:** Intent to offer help in dating violence before and after the intervention by experimental condition

Experimental group				Control group			
Pre-test	Post-test		Total	Pre-test	Post-test		Total
	Yes	No			Yes	No	
Yes	5 (36%)	2 (14%)	7 (50%)	Yes	12 (63%)	1 (5%)	13 (68%)
No	7 (50%)	0 (0%)	7 (50%)	No	4 (21%)	2 (11%)	6 (32%)
Total	12 (86%)	2 (14%)	14 (100%)	Total	16 (84%)	3 (16%)	19 (100%)

Likewise, there was not a significant change in bystander attitudes and empathy between time points (*p* > 0.05) and Cohen’s *d* indicates small to medium effect sizes for all variables (Table 4).

**Table 4:** ESPECTA-VN and empathy scale results before and after intervention for experimental group (*n* = 14)

	Pre-test (M (SD))	Post-test (M (SD))	*p* value^*^	Effect size
Bystander attitudes				
Awareness: failure to notice	3.02 (0.63)	3.19 (0.55)	0.368	0.26
Definition: failure to identify the risk	3.31 (0.82)	3.38 (0.68)	0.673	− 0.09
Responsibility: failure to assume responsibility	3.50 (0.60)	3.68 (0.56)	0.209	0.31
Plan/self-efficacy: failure due to lack of abilities	3.40 (0.96)	2.90 (0.74)	0.102	− 0.47
Action to intervene: failure due to audience inhibition effect	3.87 (0.58)	3.52 (0.70)	0.169	− 0.45
Empathy				
Empathetic consideration	3.49 (0.75)	3.57 (0.64)	0.484	− 0.16
Assuming the other’s perspective	3.21 (0.80)	3.53 (0.56)	0.116	0.46
Personal distress	3.18 (0.72)	3.27 (0.49)	0.637	− 0.19

^*^Wilcoxon’s signed-rank test for paired sample

### Intergroup comparison from pre-test to post-test

There was not a significant change in intention to help, bystander attitudes, or empathy from pre-test to post-test between groups (Table 5). A similar percentage of both EG (86%) and CG (84%) participants reported an intention to help in dating violence situations (Table 3), a non-significant difference (*p* > 0.05). Similarly, no significant differences were observed between EG and CG for empathy or bystander attitudes (*p* > 0.05).

**Table 5:** ESPECTA-VN and empathy scales before and after the intervention by experimental condition

	Experimental group (*n* = 14)		Control group (*n* = 19)		*p* value^*^
	Pre-test (M (SD))	Post-test (M (SD))	Pre-test (M (SD))	Post-test (M (SD))	
Bystander attitudes					
Awareness	3.02 (0.63)	3.19 (0.55)	3.28 (0.77)	3.45 (0.90)	0.473
Definition	3.31 (0.82)	3.38 (0.68)	3.07 (1.06)	3.37 (1.06)	0.428
Responsibility failure	3.50 (0.60)	3.68 (0.56)	3.42 (0.74)	3.48 (0.87)	0.477
Plan/self-efficacy	3.40 (0.96)	2.90 (0.74)	2.86 (1.03)	2.77 (0.94)	0.350
Action to intervene	3.87 (0.58)	3.52 (0.70)	3.45 (1.16)	3.42 (1.13)	0.326
Empathy					
Empathetic consideration	3.49 (0.75)	3.57 (0.64)	3.79 (0.74)	3.78 (0.86)	0.597
Assuming the other’s perspective	3.21 (0.80)	3.53 (0.56)	3.34 (0.50)	3.51 (0.57)	0.688
Personal distress	3.18 (0.72)	3.27 (0.49)	3.14 (0.53)	3.04 (0.85)	0.361

^*^Kruskal-Wallis test, pre- and post-test difference

## Discussion

The present study was undertaken to evaluate the efficacy of a peer- and bystander approach-based intervention for preventing dating violence by investigating its effects on intention to help, empathy, and bystander attitudes in response to dating violence in a sample of Brazilian adolescents. The hypothesis that the participants in the intervention group would intend to help more frequently than participants in the control group and have more empathy and more sympathetic bystander attitudes in response to dating violence at two and half months after the intervention was refuted. Similar results showing absence of effects of bystander interventions for dating violence prevention to improve intentions to help (Borsky et al., 2018) and willingness to help (Miller et al., 2012) were also found in other studies. However, the present data are in disagreement with other studies that have showed positive effects on intention to help (Amar et al., 2015; Miller et al., 2012; Peterson et al., 2018) and a perception of responsibility to help (Amar et al., 2015). Likewise, the lack of change in empathy runs counter to evidence that a spectator intervention boosts bystander empathy in cases of bullying prevention (Jenkins & Nickerson, 2017).

Several explanatory paths might be explored to shed light on mechanisms responsible for the failure of the intervention to produce positive short-term effects in a sample of Brazilian adolescents. First, it is possible that the failure occurred in the theory of intervention action because of the omission of core elements necessary for changing the selected outcomes. If this was the case, such an omission could have been expressed in the choice of intervention objectives and themes, such as the lack of a discussion of *when* to intervene, apart from *how* to. The inclusion of a range of bystander intervention opportunities, before, during, and after episodes of dating violence, with differing degrees of risk, could more safely equip the participants with proactive and reactive responses to violence, similar to what has been recommended in bystander interventions for situations of sexual violence (McMahon & Banyard, 2012).

Second, one could ask whether the intervention was appropriate for the developmental stage of the adolescents. Evidence from a recent systematic review and meta-analysis indicates that bystander interventions in cases of sexual abuse have more impact on youths in their first years of college than in the final years, possibly because this period provides more opportunities for affective-sexual interactions between peers and, additionally, more opportunities for bystander intervention (Kettrey & Marx, 2018). Even though these data are not derived from studies of the experience of dating, they could be considered as hypotheses for future analysis.

Third, it could be supposed that even having had an appropriate design and implementation, limitations in its evaluation might have existed, for example the sample size being too small to possess sufficient statistical power to identify changes which did occur, and an excessively short evaluation time for detecting results. This last hypothesis, related to the evaluation timing, finds support in evidence from the longitudinal analyses of a dating violence bystander intervention which showed more salient effects later (Coker et al., 2017; Coker et al., 2018), although the outcomes under consideration in these studies were distinct from the present one.

Finally, speculatively, contextual and cultural barriers to prevailing social norms around offering to help in cases of intimate partner violence may have inhibited the effects of the intervention. One prevailing social norm in Brazilian culture is to not intervene in violence between couples, expressed in the popular saying “no one gets in the middle of a fight between husband and wife” (or in Portuguese, “em briga de marido e mulher não se mete a colher”). This banalizes the violence and extends into dating relationships, resulting in significant barriers to asking for help (Njaine et al., 2011) and offering to help for adolescents to their friends involved in dating violence (Murta et al., 2014).

The absence of statistically significant effects on empathy raises questions about whether the change mechanism was adequately or sufficiently addressed in the intervention. Assuming the other’s perspective is a cognitive component of empathy which integrates this change mechanism and resembles the friendship function of self-validation. It involves the ability to recognize the other’s feelings, thereby spontaneously adopting the friend’s perspective, enabling the anticipation of reactions and behaviors. Self-validation is related to the perception that a friend is someone who listens, calms, and encourages (Souza & Hutz, 2007). In this sense, the cultivation of self-validation can be an important platform for acquiring empathetic skills that focus on assuming the other’s perspective, and vice-versa. The validation of the partner’s sentiments and thoughts can improve the resolution of couple’s conflicts and help them cope with their differences more openly and cooperatively by respecting opinions and expressing more positive sentiments in the relationship (Costa, Cenci, & Mosmann, 2016), aspects inversely related to physical and psychological aggression in dating (Cornelius, Shorey, & Beebe, 2010).

It is important to highlight that assuming the other’s perspective was a key component in the last session of this intervention. In this session, empathy skills were taught to incentivize empathetic communication and assuming the other’s perspective in response to dating violence. The adolescents were told to adopt the perspective of a potential bystander whose degree of involvement with the victim or aggressor (known or stranger) was previously determined. The bystander’s response in the face of the violent situation presented in the video was the focus of the discussion about the stages and obstacles of the bystander approach. However, this study’s data do not allow the drawing of conclusions about the dose (number of sessions offered on the empathy theme) or coverage of the change mechanism in the procedures adopted having been insufficient. Future studies might test different dosages and new procedures and examine their effects on assuming the other’s perspective and self-validation, as well as the relationships between these variables and the intention to help.

The central contributions of the present study lie firstly in the development and testing of a pioneer technology for preventing dating violence using bystander intervention between Brazilian adolescents. It is the first study in the Brazilian culture to test a more ecological intervention that moves beyond changing individuals to changing peer attitudes and skills. Thus, the present intervention combines the already existing focus on changing individual behavior (Murta et al., 2013; Murta et al., 2016; Priolo Filho, 2017) and widening the range of possibilities of potentially applicable interventions for Brazilian adolescents, once it has been improved, reassessed, and shown to be effective. This is especially relevant in the face of the paucity of preventive and educational actions for girls and boys indicated in an evaluation done by the Federal Court of Accounts (Tribunal de Contas da União) via the Maria da Penha Law (Murta et al., 2014), which regulates the fight against violence against women in Brazil. Secondly, the present study’s findings align with international ones that indicate the absence of change (Borsky et al., 2018; Moynihan et al., 2011) or mixed effects (Katz et al., 2011; Miller et al., 2012) resulting from bystander interventions for the prevention of dating violence and invite new studies capable of giving insight into the reasons for the lack of efficacy. Understanding the contexts and mechanisms that produce changes is as relevant as illuminating those that impede them.

The results of this study must be interpreted in light of its methodological limitations. First, the small sample is the main obstacle to statistical conclusion validity. The low number of participants in both groups reduced the statistical power to identify changes, if they existed. Second, the high dropout rate of participants in the post-test in both groups deserves attention. Such high attrition raises questions surrounding those adolescents who quit and those who completed the study, questions which undermine the possibility of validly interpreting the intervention results. Third, the nested structure of the data was not considered in the data analysis and future bigger school-based studies should advance in this direction. Fourth, the absence of an intention-to-treat analysis may have reduced the comparability between groups and produced bias due to not preserving the original randomization and excluding participants whose data was not available in the post-test evaluation. Claiming a non-existing effect or neglecting an existing effect (the risk in the present study) may be distortions resulting from not performing an intention-to-treat analysis. Lastly, potential selection biases might have affected the external validity given the characteristics of the setting and sample, which involved specifically adolescents in situations of risk and vulnerability and attached to a socio-professional education service promoting productive inclusion. As a result, such study design flaws preclude a definitive conclusion regarding the short-term efficacy of the intervention on the selected outcomes.

## Conclusion

The present study, centered on the evaluation of short-term efficacy of an intervention for preventing dating violence and focused on peers and the bystander approach, found no evidence for efficacy of the intervention on intention to help, empathy, or bystander attitudes. Such results should be interpreted cautiously, given that the current study design does not allow us to accurately evaluate the changes produced by the intervention. Regardless of such limitations and null effects, the study represents a starting point for research into the prevention of abusive relationships among young couples by using bystander intervention in a Brazilian context, as dating violence reaches alarming rates among Brazilian teens and given the recentness of the national production of interventions for preventing dating violence (Murta et al., 2013; Murta et al., 2016; Priolo Filho, 2017). The broader understanding of the efficacy of the present intervention is conditioned by the performance of other longitudinal efficacy studies capable of illuminating such findings, assuming the stated limitations have been overcome.

Studies that indicate evidence for null effects of interventions may offer a rich opportunity of learning from “failure” and invite enlargement of the frontiers of knowledge in prevention science (Axford, Berry, Lloyd, Wyatt, & Hobbs, 2018). Based on that, a promising agenda of research is open for future studies and intervention design as well as their evaluation. Regarding intervention design, it is critical to insert into the intervention scope detailed information about the opportunities for intervention, before, during, and after violent episodes (McMahon & Banyard, 2012) as well as the consequences of giving and not giving help (Witte, Casper, Hackman, & Mulla, 2017). It is also important to broadly discuss aspects that can affect the interpretation of these problematic events, influenced by the ambiguity related to consent, the risk involved in the situation, and the nature of the relationship between the potential victim and the potential aggressor (Burn, 2009). Additionally, it is desirable to include information for evaluating the dangerousness of each situation, spreading proper security procedures, given the high percentage of students predisposed to risky behaviors (Branch et al., 2013).

Since the social consequences and perception of peers’ norms are the main concern of adolescents when deciding whether to intervene in dating violence, they are major obstacles to taking action, preventive efforts must take into account these adolescent concerns when designing interventions based on the bystander approach (Casey, Lindhorst, & Storer, 2016). Failure due to audience effect may signal adolescents’ concern about possible negative evaluation from other bystanders. Thus, the inclusion of psychoeducational components capable of reducing potential risks for adolescent bystanders, such as space for discussion about the costs of making an improper response or looking foolish when deciding to help, can be quite useful (Burn, 2009). It is desirable as well to teach them how to cope with the fear that something might go wrong or that they might get in trouble, cause trouble, give bad advice, or even get hurt when intervening (Weisz & Black, 2008). Giving alternatives for a safe and effective intervention could be a strategy for adolescents to work out social worries stemming from fear of backlash, real or perceived risk of physical intimidation, and fear of social embarrassment (Branch et al., 2013), which could boost the intent to help and to offer help to friends and peers in dating violence situations. Finally, to boost the potential effectiveness of the bystander approach, it is crucial to insert into the intervention devices that relentlessly challenge the myth that “no one gets in the middle of in a fight between husband and wife,” deeply rooted in Brazilian culture.

Regarding intervention evaluation, it is necessary to invest in new efficacy studies using more robust research designs (Gottfredson et al., 2015). To maximize the statistical power by increasing sample size and considering other populations and different contexts is recommended (Nezu & Nezu, 2008). Including follow-up evaluations is elemental for assessing the medium- and long-term effects and to identify changing patterns in the outcomes over time, such as strengthening or weakening of changes and the moment at which a change occurs. This gains relevance upon considering that the outcomes could require more than two and a half months to manifest (Coker et al., 2017; Coker et al., 2018).

To understand the factors affecting this intervention, it is critical to evaluate, in future studies, potential mediators and moderators of its effects (Hayes, Laurenceau, & Cardaciotto, 2008). The elucidation of these mechanisms may have important implications for the design of and methods used to evaluate the intervention and be reflected in the duration and number of sessions dedicated to empathy training, for example. That said, new tests must take into account designs with variations at the time of evaluation of these factors and in dose of these components furnished (Steckler & Linnan, 2002), beyond taking measures to assure a satisfactory retention rate from the participants (Whitaker et al., 2006). If the potential effects of this intervention were realized by adolescents, in the short term, not only is a change in attitudes associated with intervening in dating violence expected, but above all, an effective mobilization of the friendship network in reducing tolerance of dating violence among peers, increasing behaviors of seeking and offering help in the mid-term, and reduction of victimization and perpetration by dating violence in the long-term as well. Hopefully, continuing research efforts in this field can combine to broaden and improve the range of services seeking to prevent intimate partner violence for Brazilian adolescents, as well as weak societal norms of tolerance toward violence which propagate daily through the national culture.

## Data Availability

Data are available from the first author.

## Abbreviations

CG: Control group
DMIRS: Davis Multidimensional Interpersonal Reactivity Scale
EG: Experimental group
ESPECTA-VN: Bystander Attitude in Dating Violence Scale
